# Alleviation of Synovial Inflammation of Juanbi-Tang on Collagen-Induced Arthritis and TNF-Tg Mice Model

**DOI:** 10.3389/fphar.2020.00045

**Published:** 2020-02-14

**Authors:** Tengteng Wang, Qingyun Jia, Tao Chen, Hao Yin, Xiaoting Tian, Xi Lin, Yang Liu, Yongjian Zhao, Yongjun Wang, Qi Shi, Chenggang Huang, Hao Xu, Qianqian Liang

**Affiliations:** ^1^Longhua Hospital, Shanghai University of Traditional Chinese Medicine, Shanghai, China; ^2^Institute of Spine, Shanghai University of Traditional Chinese Medicine, Shanghai, China; ^3^Second Ward of Trauma Surgery Department, Linyi People's Hospital, Linyi, China; ^4^Key Laboratory of Theory and Therapy of Muscles and Bones, Ministry of Education, Shanghai University of Traditional Chinese Medicine, Shanghai, China; ^5^Shanghai Institute of Materia Medica, Chinese Academy of Sciences, Shanghai, China; ^6^Department of Pathology and Laboratory Medicine, University of Rochester, Rochester, NY, United States

**Keywords:** Juanbi-Tang, rheumatoid arthritis, collagen-induced arthritis, synoviocyte, tumor necrosis factor

## Abstract

Rheumatoid arthritis (RA) is a chronic autoimmune disease that is primarily characterized by synovial inflammation. In this study, we found that a traditional Chinese decoction, Juanbi-Tang (JBT), JBT attenuated the symptoms of collagen-induced arthritis (CIA) mice and in tumor necrosis factor transgenic (TNF-Tg) mice by attenuating the arthritis index and hind paw thickness. According to histopathological staining of ankle sections, JBT significantly decreased the area of inflammation and reduced bone destruction of ankle joints in both these two types of mice. Moreover, decreased tartaric acid phosphatase-positive osteoclasts were observed in the JBT group compared with those found in the control group. We also revealed that JBT suppressed monocytes and T cells as well as the production of CCL2, CCR6, and CXCR3 ligands. We next used high-performance liquid chromatography to investigate the components and pharmacological properties of this classical herbal medicine in traditional Chinese medicine. Based on network pharmacology, we performed computational prediction simulation of the potential targets of JBT, which indicated the NF-kappa B pathway as its target, which was confirmed in vitro. JBT suppressed the production of pro-inflammatory cytokines including interleukin-6 (IL-6) and IL-8, and inhibited the expression of matrix metalloproteinase 1 in fibroblast-like synoviocytes derived from RA patients (MH7A cells). Furthermore, JBT also suppressed the phosphorylation of p38, JNK, and p65 in TNF-α-treated MH7A cells. In summary, this study proved that JBT could inhibit synovial inflammation and bone destruction, possibly by blocking the phosphorylation of NF-kappa B pathway-mediated production of proinﬂammatory effectors.

## Background

Rheumatoid arthritis (RA), one of the major contributors to disability globally (Vos et al., 2017), is characterized by synovitis, pannus formation, adjacent bone erosion, and joint destruction (Rana et al., 2018), although its etiology remains largely unclear. Among the potential cellular participants in RA, fibroblast-like synoviocytes (FLSs) play a critical role by regulating the secretion of inflammatory mediators and expression of matrix metalloproteinases (MMPs), which cause changes in chondrocyte metabolism and matrix degradation (Korb-Pap et al., 2016). Treatment targeting RA-FLSs has been recognized as a novel approach with the potential to improve clinical outcomes while having limited impact on systemic immunity (Wangyang et al., 2018; Zhang and Zhou, 2018). Tumor necrosis factor-alpha (TNF-α) acts as a primary cytokine in the pathology of RA. TNF-α can rapidly induce MMP gene expression in cultured FLSs. Its inhibitors provide other options for RA patients in whom treatment with conventional disease-modifying anti-rheumatic drugs (DMARDs) has failed (Luchetti et al., 2017; Ornbjerg, 2018). Given its immunogenicity and high cost, an increasing number of alternatives to DMARDs are being explored (Sun et al., 2018).

Juanbi-Tang (JBT), traditional Chinese herbal compounds, has been widely used for the treatment of RA in China. It has proven to relieve the symptoms of arthritis and activate joint function of patients (Li, 2011; Li and Niu, 2011; Ni and Fu, 2014). In addition, JBT has been reported to reduce serum TNF-alpha and IL-1 levels in rats with RA (Yu et al., 2015), downregulate IgG in serum (Cheng et al., 2018) and prostaglandin E receptor 4 in synovial tissue (Xu et al., 2017), and relieve acute and chronic ear inflammation in rats (Niu et al., 2018a). It was also demonstrated that JBT could regulate immune balance by reducing nature killer cells and promoting T-reg cells of CIA rats (Niu et al., 2018b), and reduce the cAMP level in T lymphocytes in rats with adjuvant arthritis (Xu et al., 2018). However, the mechanism by which JBT reduces inflammatory cytokines and chemokines warrants further research.

With the increasing investment in research on traditional Chinese herbal medicine, it has become increasingly important to determine the efficacy, toxicology, and therapeutic targets of such medicine, for which network pharmacology is a useful approach (Jiang et al., 2015).

Our study involved a search for as many as 44 active components of JBT by high-performance liquid chromatography–quadrupole time-of-flight mass spectrometry (HPLC–Q-TOF). By applying a network pharmacology method, we also enumerated the possible targets of the herbal components to look for their pharmacological effects. We further performed experiments to verify these predictions.

## Materials and Methods

### Preparation of JBT

Herbs in JBT (**Table 1**) were authenticated by a pharmacognosist of Longhua Hospital affiliated to Shanghai University of Traditional Chinese Medicine, in accordance with standard protocols. In line with standard methods of Chinese Pharmacopoeia, all crude drugs except *Cinnamomi cortex* were soaked in 12 volumes of water for 40 min and boiled for 40 min (*C. cassia* was added at 35 min). The extracts were filtered, and the filter residue was boiled in eight volumes of water for another 40 min and the solution was filtered again. Both batches of the filtrate of the drugs were mixed and concentrated to 0.557 kg/L, which were prepared for intragastric administration. The filtrate was vacuum-freeze-dehydrated, prepared to treat cells after high-speed centrifugation three times, and stored at −80°C overnight.

**Table 1:** Prescription of Juanbi-Tang (JBT).

Latin name	Amount (g)	Lot No.	Place of origin	Company
Radix angelicae pubescentis	3 g	140421-1	Hubei, China	Shanghai WanShiCheng Chinese Medicine Co. Ltd.
Notopterygium incisum	3 g	140529	Sichuan, China	Shanghai Hongqiao traditional Chinese medicine decoction pieces Co., Ltd.
Cinnamomum cassia Presl	1.5 g	140326-1	Guangxi, China	Shanghai WanShiCheng Chinese Medicine Co. Ltd.
Gentiana macrophylla Pall.	3 g	140620	Gansu, China	Shanghai Hongqiao traditional Chinese medicine decoction pieces Co., Ltd.
Angelica sinensis (Oliv.) Diels	9 g	140627	Gansu, China	Shanghai Kangqiao Chinese Herbal Medicine Co. Ltd.
Ligusticum chuanxiong Hort.	2.1 g	140518	Sichuan, China	Shanghai Kangqiao Chinese Herbal Medicine Co. Ltd.
Glycyrrhiza uralensis Fisch.	1.5 g	140616	Xinjiang, China	Shanghai Kangqiao Chinese Herbal Medicine Co. Ltd.
Caulis Piperis Kadsurae	6 g	131106-1	Yunnan, China	Shanghai WanShiCheng Chinese Medicine Co. Ltd.
Morus alba L	9 g	140624	Zhejiang, China	Shanghai Hongqiao traditional Chinese medicine decoction pieces Co., Ltd.
Olibanum	2.4 g	LY1303120	Ethiopia	Shanghai Hua Yu Pharmaceutical Co., Ltd.
Radix Aucklandiae	2.4 g	2014061004	Yunnan, China	Shanghai Hua Pu Chinese medicine decoction pieces Co., Ltd.

### Other Agents

Methotrexate (MTX, cat. #100138) was purchased from Shanghai Oriental Medicine Science and Technology Industry Co., Ltd. (Shanghai, China); bovine type II collagen (cat. #20021), Freund's complete adjuvant (CFA, cat. #7001), and Freund's incomplete adjuvant (IFA, cat. #7002) were from Chondrex (Redmond, WA, USA); eosin (Sigma, cat. #E4009-5G), hematoxylin (Sigma, cat. #H-3136), Alcian Blue (Sigma, cat. #A5268), orange G (Sigma, cat. #1936-15-8), phloxine B (Sigma, cat. #18472-87-2), ammonium aluminum sulfate (Sigma, cat. #A-2140), sodium iodate (Sigma, cat. #S-4007), tartaric acid (Sigma-vetec, cat. #87-69-4), phenol AS-BI phosphate (Sigma, cat. #1919-91-1), phosphate buffered saline (PBS) (Medicago, cat. #09-2052-100), MCP-1 polyclonal antibody (CCL2) (ABclonal, cat. #A7277), CXCR3 polyclonal antibody (ABclonal, A2939), p44/42 MAPK (Erk1/2) (137F5) (rabbit mAb, CST, cat. #4695s), recombinant anti-CD3 antibody [SP162] (Abcam, cat. #ab135372), anti-CCR6 (Abcam, cat. #ab78429), F4/80 antibody (GeneTex, cat. #GTX26640), iNOS polyclonal antibody (Thermo Fisher Scientific, cat. #PA1-036), Diaminobenzidine (DAB) Histochemistry Kit (Thermo Fisher Scientific, cat. #D22187), DAB Peroxidase (HRP) Substrate Kit (with nickel) (VECTOR, cat.#SK-4100), VECTASTAIN^®^ Elite^®^ ABC HRP Kit (VECTOR, cat. #PK-6100), goat anti-rat IgG (H+L) antibody (KPL, cat. #072-03-16-06), and goat anti-rabbit IgG (H+L) antibody (KPL, cat. #03-15-06) were also obtained. In addition, MMP-1 antibodies (cat. #ab137332) and ELISA kit (cat. #ab215083), TNFα (cat. #EMC102a), IL-1β (cat. #EMC001b), IL-6 (cat. #EMC004), IL-8 (cat. #EMC104), and ELISA kit were purchased from Neobioscience (Shanghai, China); recombinant human tumor necrosis factor (TNF-α; PeproTech, cat. #300-1A), anti-phospho-p38 (CST, cat. #4511), anti-phospho-ERK (cat. #4370), anti-phospho-JNK (CST, cat. #4255), anti-phospho-p65 (CST, cat. #3033), β-actin (CST, cat. #8457), NF-kappa B p65 (D14E12) XP^®^ Rabbit mAb (CST, cat. #8242s), ERK1/ERK2 polyclonal antibody (ABclonal, cat. #A16686), JNK1 polyclonal antibody (ABclonal, cat. #A0288), and SAPK/JNK antibody (CST, cat. #9252s); trypsin digestion solution (Leagene, cat. #IH0310), goat anti-rabbit IgG H&L (Alexa FluorR488, Abcam, cat. #ab150077), actinomycin D (Sigma, cat. #A1410), 4′,6-diamidino2-phenylindole (DAPI, Sigma, cat. #D9564), cell culture medium, fetal bovine serum, and trypsin were obtained from Gibco (Grand Island, NE, USA).

### Model Establishment and Evaluation of Collagen-Induced Arthritis

Specific pathogen-free, DBA/1J male mice (7–8-week-old) were purchased from Vital River (Beijing, China). The CIA model was established in accordance with a previously reported protocol (Brand et al., 2007). Brieﬂy, the mice were immunized intradermally with 100 mg of bovine type II collagen emulsified in complete Freund's adjuvant. To ensure a high incidence of RA induction in the CIA model, a booster immunization of bovine type II collagen emulsified in incomplete Freund's adjuvant was used at 21 d after the primary immunization. Typically, the first signs of arthritis appeared in this model at 21–28 d after immunization. CIA was considered to have successfully developed when swelling was observed in at least one paw.

### TNF-Tg Mice

The 3647 line of TNF-Tg mice in a C57BL/6 background was originally obtained from Dr. G. Kollias's lab (Keffer et al., 1991) (Institute of Immunology, Alexander Fleming Biomedical Sciences Research Center, Vari, Greece) and was maintained as heterozygotes by backcrossing with C57BL/6 mice. Non-transgenic littermates were used as aged-matched wild-type (WT) controls. This line of TNF-Tg mice carries one copy of the human TNF transgene, overexpresses human TNF-alpha, and develops erosive polyarthritis with many characteristics also observed in RA patients (Jiang et al., 2015). TNF-Tg mice develop mild ankle joint inflammation and bone erosion at 3 months old, which become more severe with aging (Li et al., 2004; Guo et al., 2009; Chen et al., 2016). Therefore, in this study, we chose 3-month-old TNF-Tg mice and WT littermates.

### Experimental Groups

Forty DBA/1J mice were randomly divided into four groups (10 mice/group): group 1, control (normal, nonimmunized and untreated); group 2, CIA-Veh [positive control, PBS, 0.2 ml/day, intraperitoneally]; group 3, CIA-MTX (MTX, 0.1 mg/kg/3 d, intraperitoneally); and group 4, CIA-JBT (JBT, 12 g/kg/day, orally). All of the mice from these groups received additional treatments between day 22 and day 50.

Seven 3-month-old TNF-Tg mice were orally administered JBT (12 g/kg) or the same volume of physiological saline once every day for 12 weeks. Seven 3-month-old WT littermates were treated with the same volume of physiological saline as a negative control. Twelve weeks later, all mice were sacrificed and their ankle joints were harvested for histological staining and data analysis.

All animal procedures were approved by Longhua Hospital Animal Ethics Committee and were performed in accordance with the Guiding Principles for the Care and Use of Laboratory Animals approved by the Animal Regulations of National Science and Technology Committee of China.

### Arthritis Assessment

After the CIA model had been successfully established, we measured the arthritis index and hind paw thickness every 5 d in a blinded manner. Each paw was scored on a scale of 0–4 by visual evaluation as follows: 0, no evidence of erythema and swelling; 1, erythema and mild swelling confined to the tarsals or ankle joint; 2, erythema and mild swelling extending from the ankle to the tarsals; 3, erythema and moderate swelling extending from ankle to metatarsal joints; and 4, erythema and severe swelling encompassing the ankle, foot, and digits, or ankylosis of the limb. The final score for each mouse was the sum of the scale score from four paws. TNF-Tg mice were measured the deformity score every week in a blinded manner for 12 weeks. Each paw was scored on a scale of 0–4 by visual evaluation as follows: 0, no deformity; 1, mild deformity; 2, obvious deformity; 3, moderate deformity, 4, severe deformity. The final score for each mouse was the sum of the scale score from four paws. Thickness of the ankle was measured separately by two individuals with digital calipers placed across the ankle joint at its widest point.

### Histopathological Examination

After final treatment, the mice were sacrificed. Ankle joints were carefully dissected and cleared of adjacent muscle, followed by being fixed in 4% paraformaldehyde at room temperature for 48 h, decalcified in 10% EDTA for 20 d, dehydrated through a series of ethanol, and embedded in paraffin. A total of 30 4-μm-thick consecutive sections from one ankle joint in the sagittal position were collected and mounted on common slides, and were divided into three levels. Each level was 40 μm from the previous level. One section from each of the three levels was subjected to hematoxylin and eosin (H&E) staining, Alcian Blue/Orange G (ABOG) staining, tartrate-resistant acid phosphatase (TRAP) staining, and immunofluorescence and immunohistochemical staining. After the full-length scan by Olympus VS120-SL, the target area of the ankle talus was analyzed with Olympus VS120 image analysis software. We traced the area of interest, which was then measured by the software (in mm^2^). The inflammatory area (multinuclear dark zone) and bone area (astragalus bone zone) were analyzed on HE-stained sections. Cartilage area (blue zone) at astragalus bone was measured on ABOG-stained sections, and TRAP-positive area was evaluated on TRAP-stained sections. The data are presented as the mean from three levels from each ankle joint sample.

### Micro-Computed Tomography (Micro-CT) Analysis

After excision from DBA/1J mice, right legs were fixed in 4% paraformaldehyde for 24 h, washed in PBS for 2 h, and then immersed in 75% ethanol, until a micro-CT (Scanco VIVA CT80, SCANCO Medical AG, Bassersdorf, Zurich, Switzerland Switzerland) scan. The X-ray tube voltage was 55 kV and tube current was 72 μA, with a pixel size of 9 μm and 200 ms integration time. The cross-sectional images were then reconstructed and realigned in 3D. A density threshold was set from 370 to 1,000 as bone using the µCT Evaluation program V6.6 (Scanco Medical AG). A stack of 340–441 cross sections was reconstructed, with an interslice distance of 1 pixel (15.6 µm), corresponding to a reconstructed height of 5.3–6.9 mm, recreating the ankle joint.

### HPLC–Q-TOF of JBT

Characterization of the components of JBT was performed by a 1260 series HPLC instrument (Agilent, Waldbronn, Germany) connected to an Agilent 6530 Q-TOF mass spectrometer (Agilent Corp., USA) equipped with Dual Agilent Jet Stream Electrospray Ionization (Dual AJS ESI). The operating parameters were optimized in both positive and negative modes, as follows: capillary voltage, 3,500 V for ESI mode and 4000 V for ESI+ mode; nozzle voltage, 500 V; fragmentor, 110 V; nebulizer, 45 psi; drying gas temperature, 300°C; drying gas flow rate, 6 L/min; sheath gas temperature, 320°C; and sheath gas flow rate, 12 L/min. Separation of compounds in JBT was carried out on an ACE Excel 3 Super C18 column (100 mm × 2.1 mm; Advanced Chromatography Technologies Ltd., Aberdeen, Scotland). The mobile phase was composed of solvent A (water containing 0.1% formic acid) and solvent B (acetonitrile containing 0.1% formic acid), with a flow rate of 0.35 ml/min. Gradient elution was performed as follows: 0–30 min, 95%–23% A; 30–40 min, 23%–15% A; 40–45 min, 15%–10% A; 45–50 min: 10%–5% A; 50–55 min, 5% A; 55–56 min: 5%–95% A; and 56–66 min, 95% A. The column temperature was maintained at 40°C. System operations and data analysis were conducted on Masshunter Workstation software (Agilent Technologies, USA).

### Network Pharmacological Analysis of JBT

The 44 active chemical components deriving from 11 components of JBT were collected by HPLC–MS. The Chinese Medicine System Pharmacology Database and Analysis Platform and Swiss Target Prediction databases were used to screen the targets of these 44 compounds. The DisGeNET database was used to find the targets related to RA. Cytoscape 3.6.0 was used to analyze the targets of JBT in the treatment of RA, and the DAVID database was used to analyze the specific mechanism of action of JBT in the treatment of RA. Twenty-four of the pharmacological components (see **Figure 3** for the chemical formulas) that satisﬁed the above conditions were selected. Then, we used Drug Bank, TCMID, Gene Cards, and STITCH databases to validate 194 RA targets to construct a compound target network using Cytoscape 3.3.0 software. To explain the participation of targets in the progression of RA, we established a network between target and function using Cytoscape 3.3.0. Multiple targets presaged an integral function of JBT in RA and shared the synergistic targets of the different compounds.

### Cell Culture

MH7A, a human RA-FLS cell line (Miyazawa et al., 1998; Jia et al., 2019), was a gift from the Chinese Academy of Sciences, Shenzhen, which had been purchased from the Riken Cell Bank (Tsukuba, Japan). Cells were maintained in Roswell Park Memorial Institute 1640 medium (Hyclone, USA), supplemented with 10% fetal bovine serum (Gibco, USA) and penicillin/streptomycin (1:100; Sigma, St. Louis, MO), in a humidified atmosphere of 95% air and 5% CO_2_ in an incubator at 37°C.

### Cell Viability Assay

Cell viability was determined using the CCK-8 assay. Briefly, MH7A cells were seeded in 96-well culture plates at 5000 cells/well for 72 h and then treated with various concentrations of JBT (0, 0.25, 0.5, 1, and 2 mg/ml) for 24 h. Then, CCK-8 (Dojindo, Japan) was added and incubated at 37°C for 1.5 h. The optical density was detected at 450 nm with a microplate reader (BioTek Synergy 3).

### Quantitative Real-Time PCR Analysis

MH7A cells were pretreated with various concentrations of JBT lyophilized powder (0, 0.5, 1, and 1.5 mg/ml) for 2 h, and then incubated for another 24 h with or without stimulation by 10 ng/m TNF-α. Total RNA was extracted using Trizol reagent, in accordance with the manufacturer's instructions, and each sample was reverse-transcribed using the cDNA synthesis kit, in accordance with the manufacturer's protocol. Real-time PCR analysis was performed using SYBR Green PCR Premix Ex Taq II reagents on a CFX96 real-time system (Thermo, USA). Relative gene expression was calculated by the ^−ΔΔ^Ct method. The sequence of primers (forward and reverse) was as follows: actin, 5′-CGTTGACATCCGTAAAGACC-3′ and 5′-TAGGAGCCAGAGCAGTAATC-3′; IL-6, 5′ -TGTATGAACAACGATGATGCACTT-3′ and 5′-ACTCTGGCTTTGTCTTTCTTGTTATCT-3′; IL-8, 5′-GGTGCAGTTTTGCCAAGGAG-3′ and 5′-TTCCTTGGGGTCCAGACAGA-3′; and MMP-1, 5′-CTCAATTTCACTTCTGTTTTCTG-3′ and 5′-CATCTCTGTCGGCAAATTCGT-3′. The efficiencies of the primers were 90%-95%. The PCR reaction system was 20 µl: SYBR 10 µl, water 7 µl, cDNA 1 µl, front and rear primers 1 µl. The thermocycling condition: 95°C for 5 min, 95°C for 30 sec→60°C for 30 sec→72°C for 40 sec, 30 cycles, 72°C for 7 min, and stored at 4°C.

### Enzyme-Linked Immunosorbent Assay

MH7A cells were seeded in six-well plates (1*10^6^ cells/well) for 24 h, and then pretreated or left untreated with the indicated concentrations of JBT for 2 h, followed by incubation for another 24 h with or without TNF-α (10 ng/ml). Cell culture supernatants were collected and stored at −80°C until analysis. The concentrations of the cytokines in culture supernatants were determined by ELISA using a commercial kit, in accordance with the manufacturer's instructions.

### Western Blot Analysis

MH7A cells were treated with or without TNF-α (10 ng/ml) in the presence or absence of JBT (0.5, 1, and 1.5 mg/ml) for 24 or 0.5 h. The protein was collected, and 30 μg of it from each sample was separated by 10% SDS-PAGE and transferred to a polyvinylidene fluoride membrane. After blocking with 5% bovine serum albumin in TBST at room temperature for 1 h, the membranes were incubated with the corresponding primary antibodies overnight at 4°C. After washing with TBST three times, the membranes were incubated with the secondary antibodies. Proteins were scanned and analyzed by the chemiluminescence system and autoradiography.

### Statistical Analysis

The data are presented as mean ± standard deviation (SD) from at least three separate experiments. Statistical analysis was performed using GraphPad Prism 7.0 software (GraphPad, La Jolla, CA, USA). One-way ANOVA followed by Dunnett's t-test was used to determine the significance of differences between groups. In cases of three groups treated at different times points, we applied two-way ANOVA for comparison. We used Fisher's test in the analysis of categorical data. *P <* 0.05 was considered statistically significant.

## Results

### JBT Clearly Attenuated Symptoms, Synovitis, Cartilage, and Bone Damage of CIA Mice

**Figure 1:**
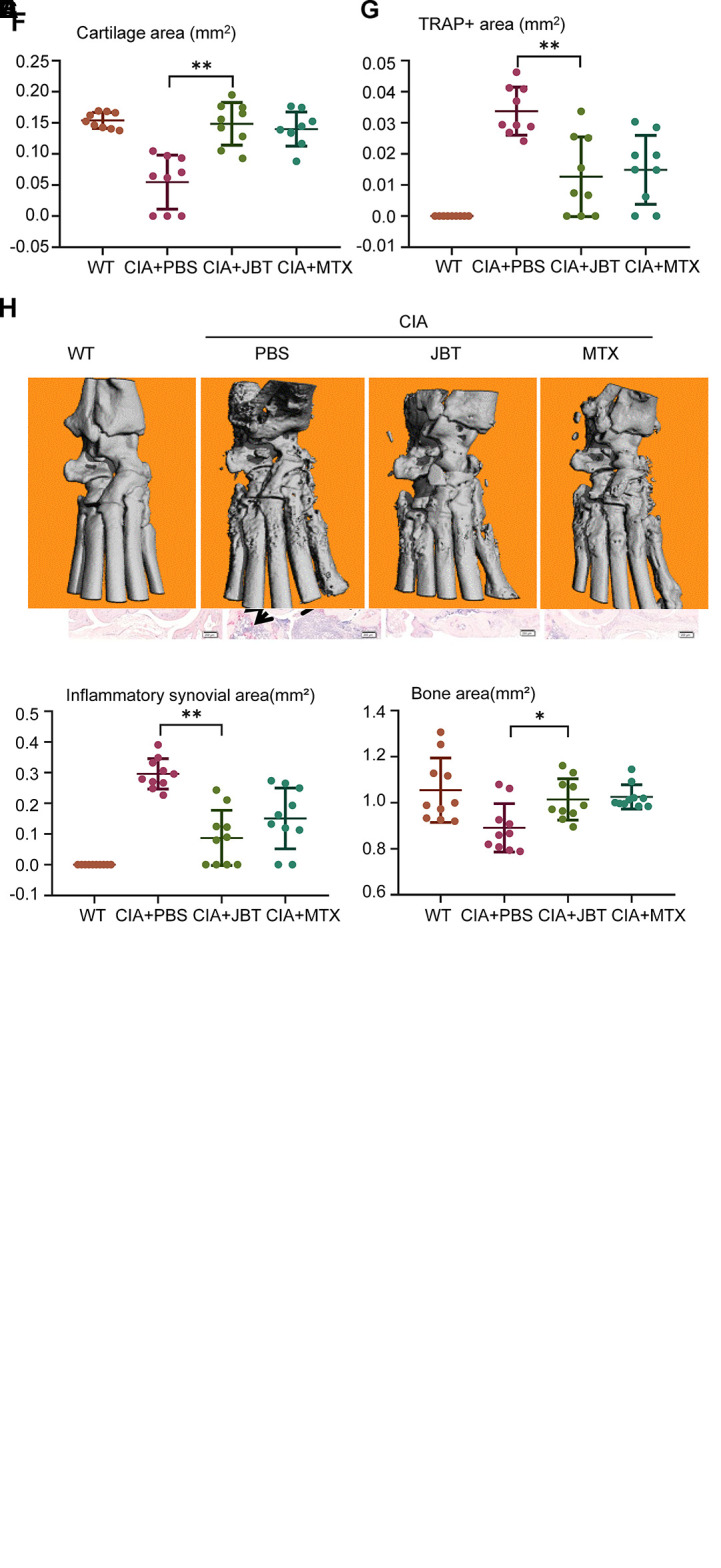
Juanbi-Tang (JBT) clearly attenuated symptoms, synovitis, cartilage, and bone damage of collagen-induced arthritis (CIA) mice. The arthritis index **(A)** and hind paw thickness **(B)** of CIA mice were scored and recorded every 5 d in a blinded manner. **(C)** Hematoxylin–eosin staining, ABOG staining, and TRAP staining were used to analyze the ankle joints of WT and CIA mice after treatment for 12 weeks. Bar, 200 μm. **(D)** Histomorphometric assessment of inflammatory synovial volume area. **(E)** Histomorphometric assessment of bone area. **(F)** Histomorphometric assessment of cartilage area. **(G)** Histomorphometric assessment of TRAP+ osteoclast area. **(H)** Micro-CT of ankle joints. Results are shown as mean ± SEM (n = 10). *p < 0.05, **p < 0.01 versus CIA+PBS group.

To investigate the therapeutic effects of JBT on RA *in vivo*, we established a CIA mouse model. Compared with the WT group, CIA mice showed higher scores for the arthritis index and greater hind paw thickness. JBT treatment had improved the function of ankle by attenuating the arthritis index (**Figure 1A**) and hind paw thickness (**Figure 1B**). HE, ABOG, and TRAP staining at ankle joints was scanned using an Olympus VS120-SL (**Figure 1C**). Treatment of mice with JBT was initiated at 22 d after the primary immunization. Both JBT and MTX treatments had positive effects on attenuating the inflammatory area (**Figure 1D**) and restoring bone (**Figure 1E**) and cartilage area (**Figure 1F**) of CIA mice. JBT treatment significantly reduced the number of osteoclasts with TRAP-positive staining (**Figure 1G**). Micro-CT showed that the JBT group, as well as the MTX group, suffered less bone damage in their ankle joints than the control group (**Figure 1H**). These results indicated that JBT effectively inhibited the development of arthritis in CIA mice.

### JBT Attenuated Symptoms, Inflammatory Synovial Volume and Bone Erosion at Ankle Joint of TNF-Tg Mice

**Figure 2:**
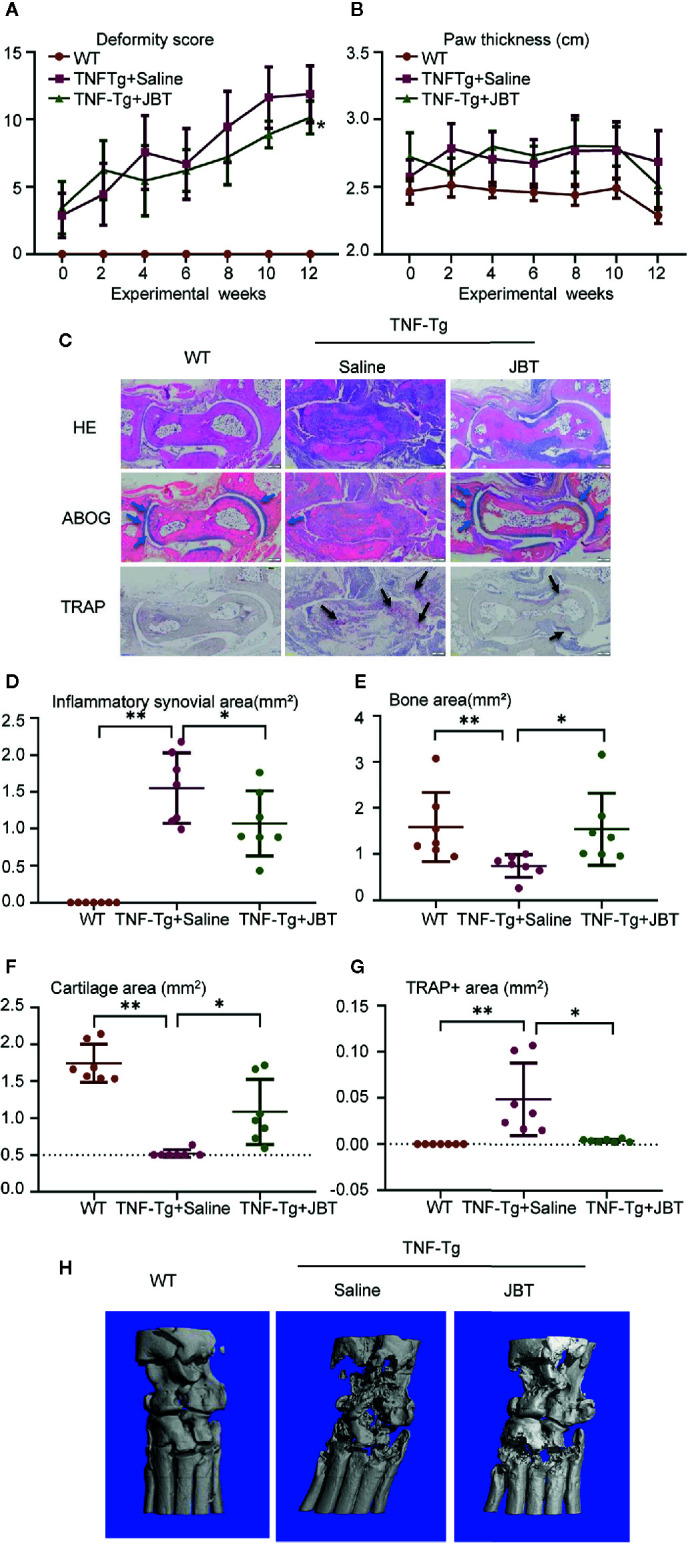
Juanbi-Tang (JBT) attenuated symptoms, inflammatory synovial volume and bone erosion at ankle joint of tumor necrosis factor transgenic (TNF-Tg) mice. The deformity score **(A)** and hind paw thickness **(B)** of TNF-Tg mice and WT littermates were scored and recorded every week in a blinded manner. **(C)** Hematoxylin–eosin staining, ABOG staining, and TRAP staining were used to analyze the ankle joints of WT and TNF-Tg mice, which were treated with saline or JBT for 12 weeks. Bar, 200 μm. **(D)** Histomorphometric assessment of inflammatory synovial volume area. **(E)** Histomorphometric assessment of bone area. **(F)** Histomorphometric assessment of cartilage area. **(G)** Histomorphometric assessment of TRAP+ osteoclast area. **(H)** Micro-CT of ankle joints. Results are shown as mean ± SEM, for 6–8 legs per group. Three sections per mice were analyzed. **p < 0.01 versus TNF-Tg + saline, *p < 0.05 versus TNF-Tg + saline.

To investigate the effect of JBT on arthritis, TNF-Tg mice were orally administered JBT once a day for 12 weeks. Compared with the WT group, TNF-Tg mice showed higher deformity score and greater hind paw thickness. JBT treatment had improved the function of ankle by attenuating the deformity score (**Figure 2A**) and hind paw thickness (**Figure 2B**). HE, ABOG, and TRAP staining at the ankle joints indicated normal integrity of the ankle joint without synovial inflammation and bone erosion in WT mice (**Figure 2C**). In TNF-Tg mice, there was a large amount of synovial inflammation in the ankle joint, with the presence of a large number of TRAP-positive osteoclasts, and severe bone erosion was observed at the ankle joint, while cartilage with Alcian Blue-positive staining almost disappeared. JBT treatment significantly reduced the inflammatory synovial volume, bone erosion (**Figures 2D, E**), and osteoclasts positively stained for TRAP (**Figure 2F**), while increasing cartilage area at the ankle joints (**Figure 2G**). Micro-CT showed that the JBT group suffered less bone damage in the ankle joints (**Figure 2H**). These results indicated that JBT effectively inhibited the development of arthritis in TNF-Tg mice.

### JBT Suppressed Inflammatory Cell Infiltration and the Production of CCL2, CXCR3, and CCR6

**Figure 3:**
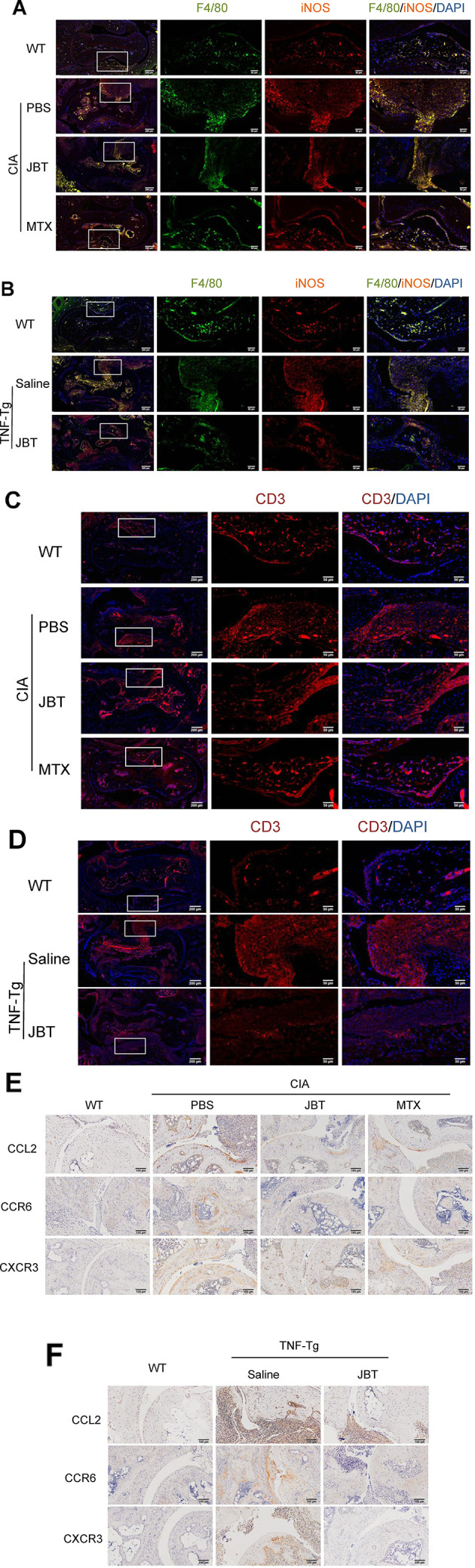
Juanbi-Tang (JBT) suppressed inflammatory cells and production of CCL2, CXCR3, and CCR6. **(A)** Immunofluorescence staining with F4/80/iNOS marking macrophages of collagen-induced arthritis (CIA) mice. **(B)** Immunofluorescence staining with F4/80/iNOS marking macrophages of tumor necrosis factor transgenic (TNF-Tg) mice and WT littermates. **(C)** Immunofluorescence staining with CD3 labeling T cells of CIA mice. **(D)** Immunofluorescence staining with CD3 labeling T cells of TNF-Tg mice and WT littermates. **(E)** Immunohistochemical staining with CCL2, CXCR3, and CCR6 of CIA mice. **(F)** Immunohistochemical staining with CCL2, CXCR3, and CCR6 of TNF-Tg mice and WT littermates.

To further investigate the effect of JBT on inflammatory cell infiltration, immunofluorescence staining was performed to mark macrophages with F4/80/iNOS and T cells with CD3. Immunohistochemical staining was performed to label CCL2, CXCR3 and CCR6. The results showed that JBT inhibited F4/80/iNOS+ macrophages (**Figures 3A, B** and **Supplementary 5A, B**) and CD3+ T cells (**Figures 3C, D**) in the ankle joints of CIA and TNF-Tg mice, and reduced the expression of CCL2, CXCR3, and CCR6 (**Figures 3E, F** and **Supplementary 5C, D**).

### HPLC–Q-TOF Detected 44 Compounds in JBT Decoction

**Figure 4:**
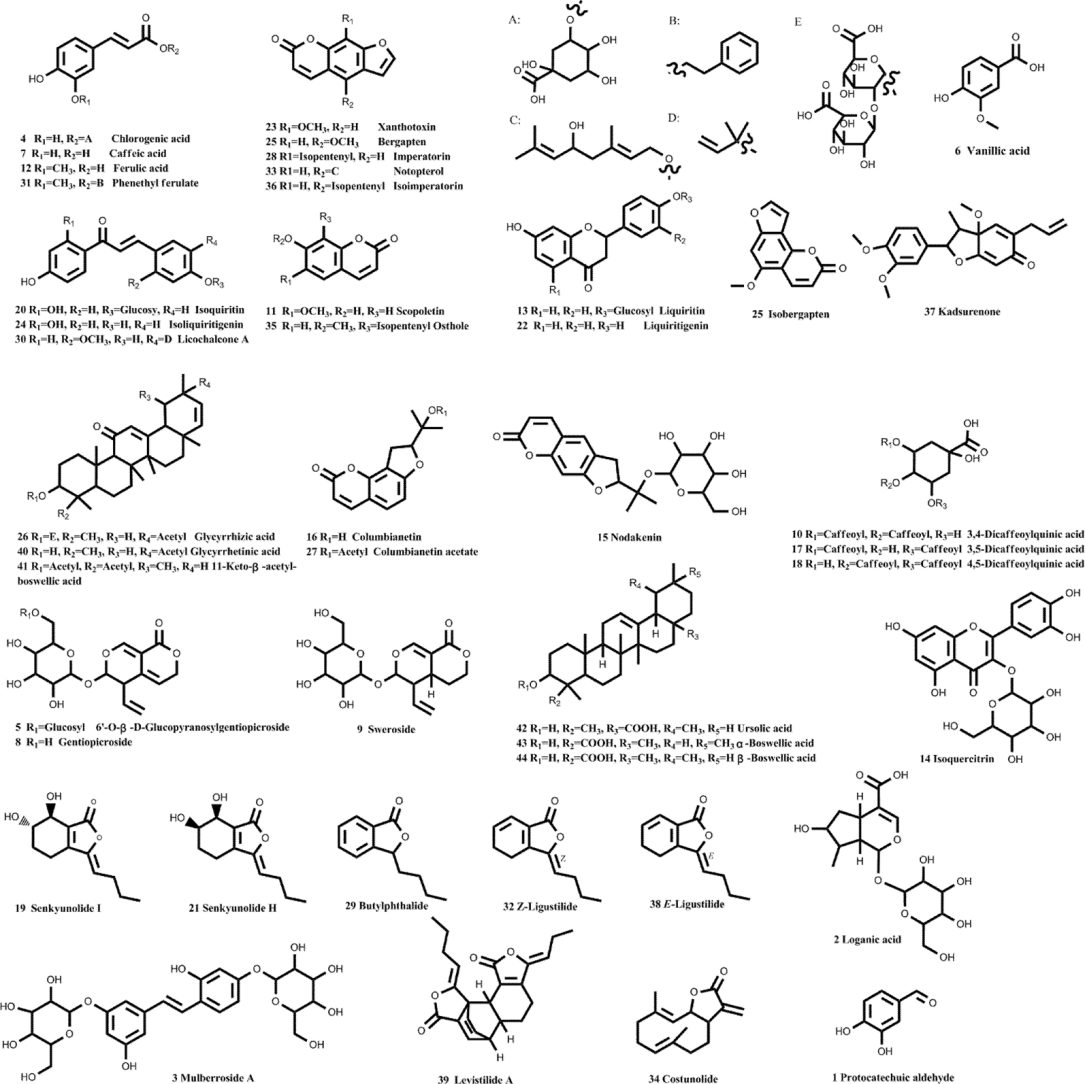
Chemical structures of 44 identified components in JBT. With the aid of accurate masses measured by HPLC–Q-TOF, a total of 44 detected peaks were identified in Juanbi-Tang (JBT) decoction, as shown in **Table 2** and **Figure 4**. Among them, 24 compounds were definitively identified by comparison with the standard compounds, while the remaining 20 compounds were tentatively characterized according to their formula, retention time, and fragmentation patterns.

With the aid of accurate masses measured by HPLC–Q-TOF, a total of 44 detected peaks were identified in the JBT decoction, as shown in **Table 2**. Among them, there were 24 compounds (**Figure 4** and **Supplementary 4**) definitively identified by comparison with the standard compounds, while the remaining 20 compounds were tentatively characterized according to their formula, retention time, and fragmentation patterns.

**Table 2:** The mass information and source of identified compounds in Juanbi-Tang (JBT) by high-performance liquid chromatography quadrupole time-of-flight mass spectrometry (HPLC–Q-TOF).

No	Rt (min)	Identification	Formula	Mass	[M+H]^+^	[M-H]^-^	Error (ppm)	Source
**1**	5.62	Protocatechuic aldehyde	C_7_H_6_O_3_	138.031		137.0242	-1.8	CNM
**2**	6.23	Loganic acid*	C_16_H_24_O_10_	376.163		375.1291	-1.6	RGM
**3**	6.93	Mulberroside A	C_26_H_32_O_14_	568.1792	569.1865		2.3	MBT
**4**	7.03	Chlorogenic acid*	C_16_H_18_O_9_	354.095	355.1025		0.4	NRR, APR
**5**	7.23	6'-O-β-D-Glucopyransylgentiopicroside	C_22_H_30_O_14_	518.164	519.1703		1.3	RGM
**6**	7.33	Vanillic acid	C_8_H_8_O_4_	168.042	169.0492		-1.4	NRR, ALS
**7**	7.53	Caffeic acid*	C_9_H_8_O_4_	180.042	181.0498		0.9	ALS
**8**	8.04	Gentiopicroside*	C_16_H_20_O_9_	356.109	357.1166		-3.6	RGM
**9**	8.24	Sweroside*	C_16_H_22_O_9_	358.123	359.134		0.8	RGM, CNM
**10**	8.75	3,4-Dicaffeoylquinic acid	C_25_H_24_O_12_	516.1268		515.119	-1.0	NRR
**11**	10.06	Scopoletin*	C_10_ H_8_O_4_	192.042	193.0495		-0.4	APR, CNM
**12**	10.26	Ferulic acid*	C_10_ H_10_O_4_	194.058	195.0652		0.2	NRR, RGM ALS, LTW APR
**13**	10.36	Liquiritin*	C_21_H_22_O_9_	418.128	419.1353		5.0	GRR
**14**	10.57	Isoquercitrin*	C_21_H_20_O_12_	464.095	465.105		-1.9	MBT
**15**	10.97	Nodakenin*	C_20_H_24_O_9_	408.144	409.1506		3.8	NRR
**16**	11,57	Columbianetin	C_14_H_14_O_4_	246.089	247.0964		-0.9	APR
**17**	11.58	3,5-Dicaffeoylquinic acid	C_25_H_24_O_12_	516.1268		515.1188	-1.4	NRR
**18**	12.18	4,5-Dicaffeoylquinic acid	C_25_H_24_O_12_	516.1268		515.1184	-2.1	NRR
**19**	12.69	Senkyunolide I*	C_12_H_16_O_4_	224.105	225.1124		1.7	LTW
**20**	13.09	Isoliquiritin	C_21_H_22_O_9_	418.127	419.1334		1.4	GRR
**21**	13.60	Senkyunolide H	C_12_H_16_O_4_	224.107	225.1118		-1.3	LTW
**22**	13.90	Liquiritigenin*	C_15_H_12_O_4_	256.074	257.0809		1.1	GRR
**23**	16.52	Xanthotoxin*	C_12_H_8_O_4_	216.042	217.0495		-0.2	NRR, APR
**24**	17.84	Isoliquiritigenin*	C_15_H_12_O_4_	256.073	257.0805		-2.2	GRR
**25**	17.94	Bergapten/ Isobergapten	C_12_H_8_O_4_	216.042	217.0495		-0.2	NRR, APR
**26**	18.75	Glycyrrhizic acid*	C_42_H_62_ O_16_	822.405	823.411		0.9	GRR
**27**	20.16	Columbianetin acetate*	C_16_H_16_O_5_	288.1	289.1071		0.2	APR
**28**	21.68	Imperatorin	C_16_H_14_O_4_	270.089	271.0967		0.6	NRR, APR
**29**	21.98	Butylphthalide*	C_12_H_14_O_2_	190.099	191.1064		0.2	LTW
**30**	22.99	Licochalcone A*	C_21_H_22_O_4_	338.152	339.1595		1.9	GRR
**31**	23.80	Phenethyl ferulate	C_18_H_18_O_4_	298.121	299.1281		2.1	NRR
**32**	23.90	Z-ligustilide	C_12_H_14_O_2_	190.099	191.1066		-0.5	ALS
**33**	24.10	Notopterol*	C_21_H_22_O_5_	354.147	355.1543		1.1	NRR
**34**	24.30	Costunolide*	C_15_H_20_O_2_	232.146	233.1536		0.1	ALR
**35**	24.40	Osthole*	C_15_H_16_O_3_	244.111	245.1177		2.7	NRR, APR
**36**	25.01	Isoimperatorin*	C_16_H_14_O_4_	270.089	271.0965		0.1	NRR, APR
**37**	25.31	Kadsurenone	C_21_H_24_O_5_	356.1623	357.1701		1.0	PKC
**38**	27.43	E-ligustilide	C_12_H_14_O_2_	190.099	191.1066		-2.7	ALS
**39**	29.86	Levistilide A*	C_24_H_28_O_4_	380.199	381.206		0.6	ALS
**40**	31.40	Glycyrrhetinic acid	C_30_H_46_O_4_	470.337		469.3303	-4.6	GRR
**41**	36.22	11-Keto-β-acetyl-boswellic acid*	C_32_H_48_O_5_	512.353	513.358		2.4	FKC
**42**	37.53	Ursolic acid	C_30_H_48_O_3_	456.361	457.3679		1.8	RGM
**43**	41.52	α-boswellic acid	C_30_H_48_O_3_	456.359		455.3515	-3.3	FKC
**44**	46.55	β-boswellic acid	C_30_H_48_O_3_	456.359		455.3515	-3.3	FKC

*Identified with the reference compounds.

Herb medicine. NRR, Notopterygii Rhizoma et Radix; APR, Angelicae Pubescentis Radix; RGM, Radix Gentianae Macrophyllae; LTW, Ligusticum wallichii; ALS, Angelica sinensis; PKC, Piperis Kadsurae Caulis; MBT, Mulberry twig; ALR, Aucklandiae radix; FKC, Piperis Kadsurae Caulis; GRR, Glycyrrhizae radix et rhizome; CNM, Cinnamon.

### Network of 44 Potential Compounds Predicted to Have 194 Potential Targets

**Figure 5:**
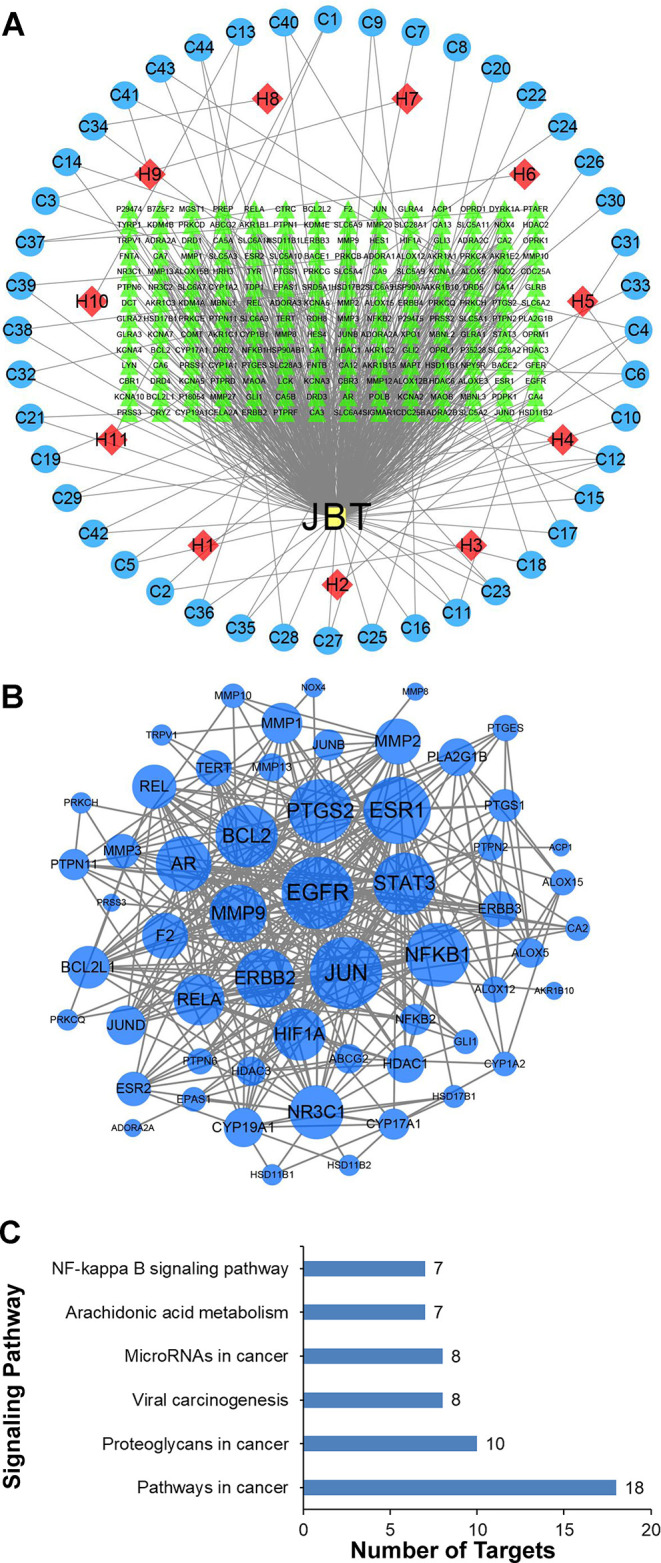
Network of 44 potential compounds predicted to have 194 potential targets. **(A)** Network of compounds in Juanbi-Tang (JBT) and their potential targets. JBT (yellow rectangle), JBT 11 herbs (H, red diamond), and network of 44 potential compounds (C, blue ellipse) predicted to have 194 potential targets (green triangle). **(B)** Target network. A node represents a protein, and the size of the nodes is proportional to the degree of the node. **(C)** Relative pathways were analyzed according to the targets.

We obtained the SMILE number of 44 compounds through the PubChem database (https://pubchem.ncbi.nlm.nih.gov/); then, we used the Swisst Target Prediction database (http://swisstargetprediction.ch/) to query the target sites of the 44 compounds. After removing the duplicates, a total of 194 targets were obtained. After querying the JBT target in the Swiss Target Prediction database, the protein interaction network related to JBT was constructed with the STRING database. Using DisGeNET (http://www.disgenet.org/) database, we found 1,848 genes related to RA. Network of compounds in JBT and their potential targets. JBT (yellow rectangle), JBT 11 herbs (H, red diamond), and network of 44 potential compounds (C, blue ellipse) predicted to have 194 potential targets (green triangle). It comprised 252 nodes and 702 edges, as shown in **Figure 5A**. We use the DAVID database to analyze the GO enrichment and KEGG pathways of JBT genes in RA. To make the research more targeted, we used the JBT targets applied as queries in DisGeNET to map the RA genes, and constructed the common genes through the STRING database to obtain the *in vivo* network of responses of RA to JBT. The JBT protein interaction network was visualized in Cytoscape 3.6.0 software (**Figure 5B**). Each protein was represented by a node and the size of the nodes is proportional to the degree of the node. Relative pathways were analyzed according to the targets. As shown in **Figure 5C**, NF-kappa B pathways was one of the most relative pathways. More specific targets were marked with pentagram (**Supplementary 1**).

### JBT Suppressed the mRNA and Protein Expression of MMP-1, IL-6, and IL-8 in TNF-α-Induced MH7A Cells

**Figure 6:**
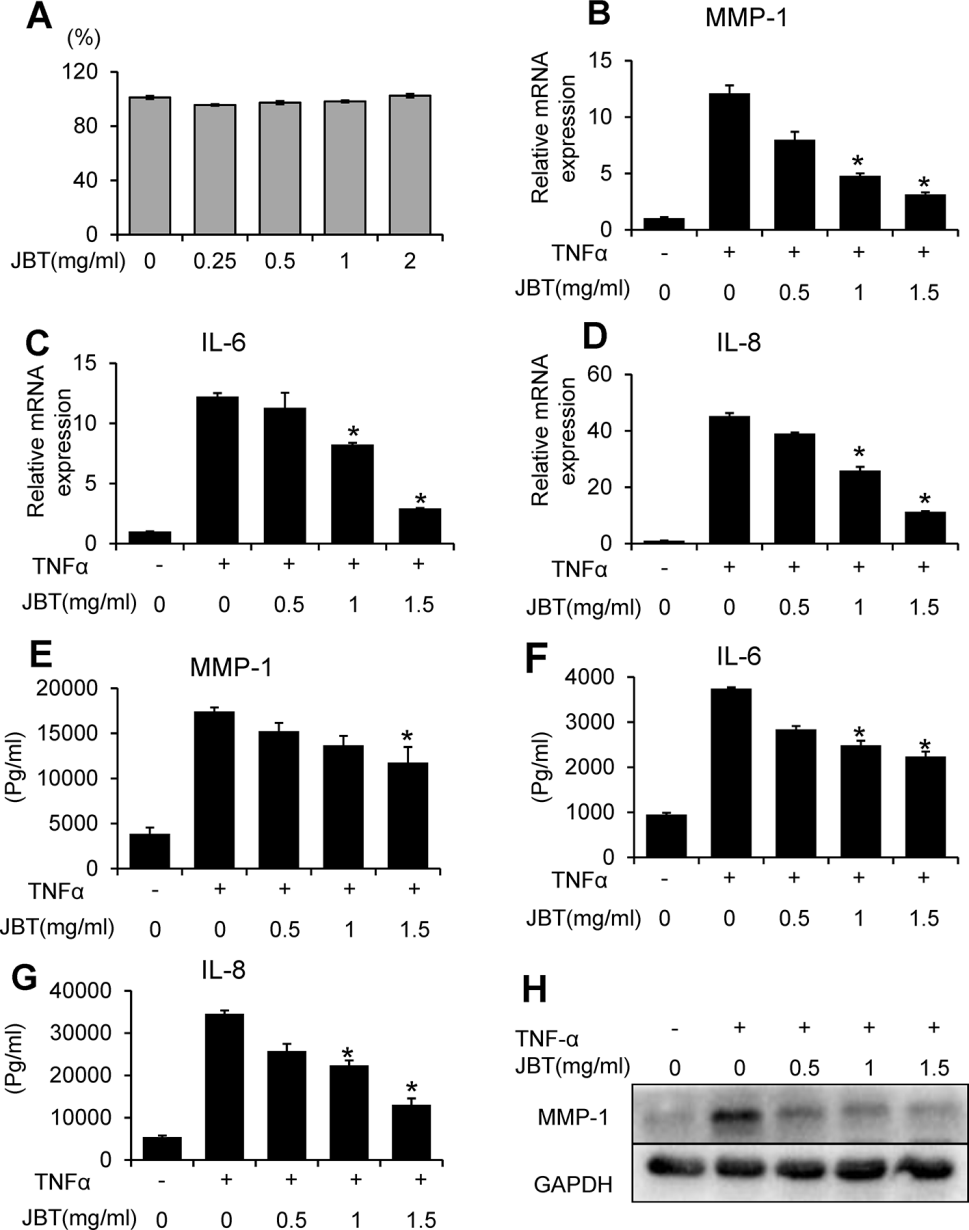
Juanbi-Tang (JBT) suppressed the mRNA and protein expression of MMP-1, IL-6, and IL-8 in TNF-α-induced MH7A cells. **(A)** CCK-8 assay of MH7A cells after treatment with different concentrations of JBT for 0, 24, 48, and 72 h. **(B–D)** The mRNA levels of MMP-1, IL-6, and IL-8 were determined using qPCR. **(E–G)** ELISA assay of MMP-1, IL-6, and IL-8. **(H)** Western blot analysis of MMP-1. Representative images are shown from three independent experiments. Results are presented as mean ± SEM of three independent experiments. *p < 0.05, versus group treated with TNF-α alone.

To assess the potential cytotoxicity of JBT, cell viability was evaluated by CCK-8 assay. As shown in **Figure 6A**, JBT at concentrations in the range of 0.25–2 mg/ml did not affect cell viability after incubation for 72 h. To determine the protective effect of JBT against the expression of inflammatory cytokines, MH7A cells were incubated for 24 h with TNF-α. TNF-α significantly increased IL-6, IL-8, and MMP-1 expression at the mRNA level (**Figures 6B–D**) and the levels excreted in conditioned medium (**Figures 6E–G**). JBT significantly decreased the mRNA and protein expression of MMP-1 in TNF-α-induced MH7A cells in a dose-dependent manner, compared with that in the control group treated with TNF-α alone (**Figure 6H**). However, JBT did not show a significant effect on the mRNA expression of MMP-3 (**Supplementary 2**). Furthermore, JBT inhibited osteoclastogenesis (**Supplementary 3**).

### JBT Inhibited TNF-α-Induced Activation of NF-Kappa B Pathways

**Figure 7:**
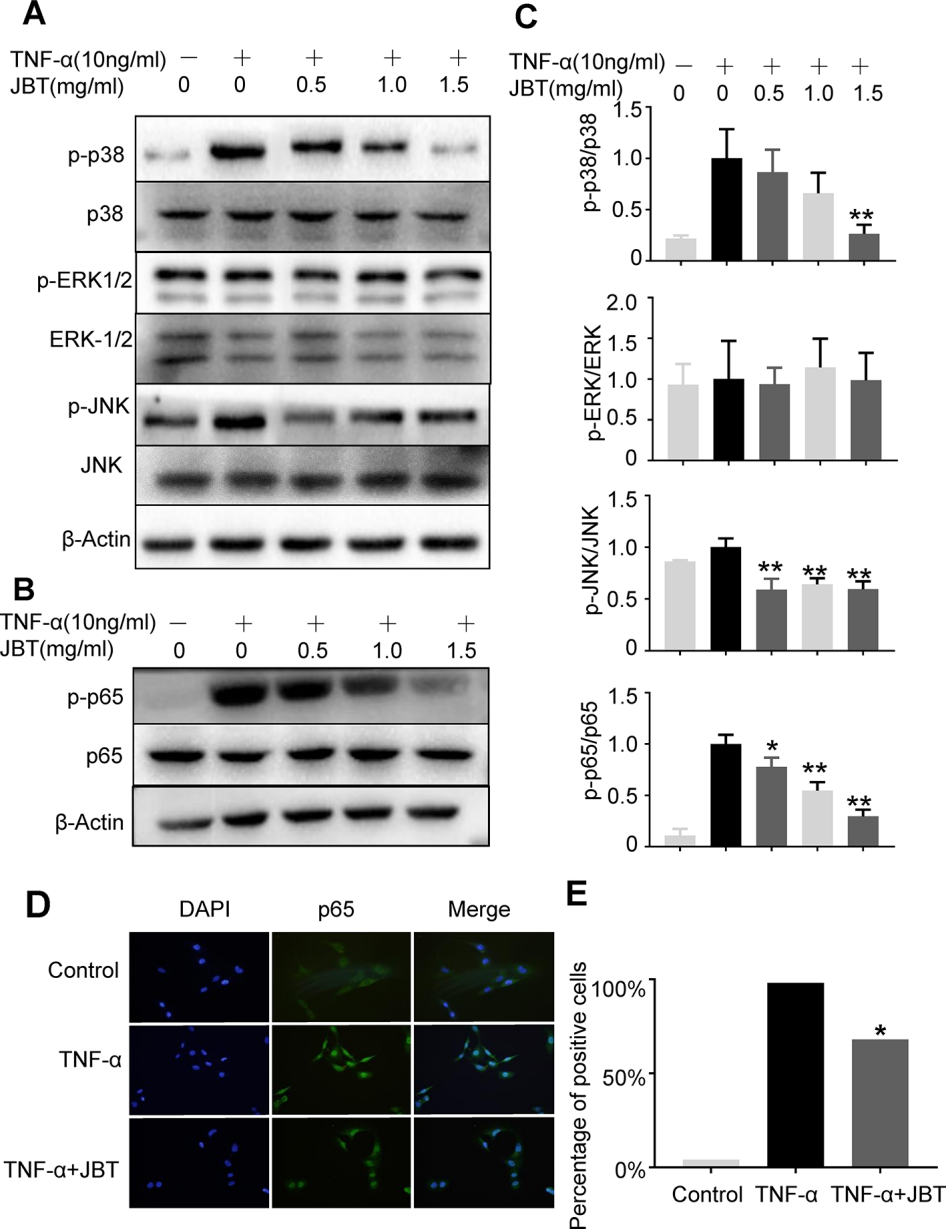
JBT inhibited TNFα-induced activation of NF-kappa B pathways. **(A-C)** The protein levels and phosphorylation levels of p38, ERK, JNK, and p65 of MH7A cells pretreated with JBT or not were determined by Western blot analysis. The total and phosphorylated levels of p38 and p65 were analyzed by Western blot analysis. Representative images are shown from three independent experiments. *p < 0.05, **p < 0.01 versus group treated with TNF-α alone. **(D)** Immunoﬂuorescence assay of p65 in the nucleus of MH7A cells. **(E)** Assessment of the percentage of cells positive for p65 in the immunoﬂuorescence assay.

To further investigate the mechanism by which JBT inhibits the production of inflammatory cytokines, we examined its effect on the NF-kappa B-mediated pathways in TNF-α-induced MH7A cells. MH7A cells were pretreated with the indicated concentrations of JBT for 2 h, before stimulation with TNF-α (10 ng/ml) for another 30 min. Then, the protein levels and phosphorylation levels of p38, ERK, JNK, and p65 were determined. We found that the phosphorylation of p38, JNK, and p65 was markedly increased after TNF-α stimulation. JBT treatment significantly inhibited the phosphorylation of p38, JNK, p65 dose-dependently (0.5, 1, and 1.5 mg/ml, **Figures 7A–C**). We also used immunoﬂuorescence analysis to determine the intracellular localization of p65. We pretreated MH7A cells with JBT (1.0 mg/ml) for 2 h or left them untreated, and then exposed them to TNF-α (10 ng/ml) for 0.5 h. The immunoﬂuorescence assay revealed that JBT signiﬁcantly reduced the level of p65 in the nucleus of MH7A cells (**Figures 7D, E**).

## Discussion

In this study, we demonstrated that JBT could effectively inhibit joint symptoms, synovial inflammation, and joint destruction in CIA and TNF-Tg mice. We also revealed that JBT suppressed monocytes and T cells as well as the production of CCL2, CCR6, and CXCR3 ligands. *In vitro* data indicated that JBT could also profoundly inhibit the TNF-α-induced production of IL-6, IL-8, and MMP-1 in cultured MH7A cells by downregulating p65 NF-kappa B pathways, as well as probably by the downregulation of p38-MAPK pathways.

JBT was originally reported in a book from the Qing dynasty called *Yi Xue Xin Wu* (“Medical Reflections”), consisting of 11 herbs: Notopterygii Rhizoma et Radix, Angelicae Pubescentis Radix, Radix Gentianae Macrophyllae, *Ligusticum wallichii* (LTW), *Angelica sinensis* (ALS), Piperis Kadsurae Caulis, mulberry twig (MBT), Aucklandiae radix (ALR), frankincense (FKC), Glycyrrhizae radix et rhizome, and cinnamon (CNM). Primary studies have shown that JBT could be used for the treatment of chronic lower back pain, external humeral epicondylitis, synovitis of the hip in children, ankylosing spondylitis, gout, and other bone diseases (Li, 2011; Li and Niu, 2011; Ni and Fu, 2014). It could also reduce inflammatory cytokines such as TNF-α and IL-1 in serum of model rats induced by Freund's adjuvant (Yu et al., 2015). Against this background, we adopted two animal models so as to verify the effect of JBT during the early stage of RA. One is the most widely accepted, the CIA mouse model, and the other is the TNF-Tg mice model with TNF-α overexpression, both of which showed clear joint inflammation, similar to RA, before undergoing treatment. We found that JBT significantly reduced joint synovitis and the articular cartilage and bone defects in both animal models, thereby clarifying its efficacy *in vivo*. In other words, it was found that JBT can effectively reduce RA joint inflammation.

To clarify the pharmacological mechanism of JBT, we employed HPLC to detect its active components. We found 44 components in it, for the first time. To further analyze the mechanisms of action of these components, we then applied a network pharmacological method. Most previous studies on the mechanism of action of JBT tended to obtain the effective components based on retrieval of the associated literature from databases. For the first time, we here instead performed this based on the components detected by HPLC, which boosted the objectivity and credibility of our experimental results. Based on the network pharmacological analysis, we found that the targets of JBT components are mainly concentrated in the NF-kappa B pathway, as well as in cancer pathways, cancer proteoglycans, cancer microRNAs, viral carcinogenesis, and arachidonic acid metabolism.

Next, we conducted *in vitro* experiments for exploring the underlying mechanism. To simulate the *in vivo* environment of TNF-Tg mice and the high expression of TNF-α in the lesion area of RA, in the current study, we treated synovial cells with TNF-α (10 ng/ml) to observe the changes of inflammatory factors produced by them upon inflammatory stimulation. We found that JBT could also inhibit the TNF-α-induced production of IL-6, IL-8, and MMP-1 in synovial cells, restoring the structure of joints.

IL-6 is highly expressed in fibroblasts during the early stage of RA (Filer et al., 2017) and its inhibitor has started being used for clinical treatment (McInnes and Schett, 2017), as well as TNF-α. IL-8 also acts as a marker of synovitis (Alam et al., 2017). Among the compounds in JBT, ferulic acid was reported to have ameliorative potential for mitigating the effects of IL-6 associated with vincristine-induced painful neuropathy (Vashistha et al., 2017). Chlorogenic acid combined with chondrocytes on an alginate scaffold decreased MMP expression and improved the recovery of damaged articular cartilage (Cheng et al., 2018). Caffeic acid repressed IL-6 and TNF-α in RA-FLS by blocking the phosphorylation of IκB and IκB kinase (Wang et al., 2017). Moreover, sweroside inhibited IL-1β-stimulated MMP-1, MMP-3, MMP-13, and ADAMTS-5 mRNA expression in rat articular chondrocytes by suppressing NF-kappa B and mTORC1 signaling (Zhang et al., 2019). Furthermore, ursolic acid reduced the incidence and severity of CIA-induced arthritis, accompanied by the decreased expression of TNF-α, IL-1β, IL-6, IL-21, and IL-17 in arthritic joints (Baek et al., 2014). Osthol was also shown to inhibit MMP-1, MMP-3, MMP-13, IL-6, and TNF-α in IL-1β-stimulated SW982 cells, as well as NF-kappa B and MAPK pathways (Xu et al., 2018). Although much research on the ingredients of JBT has been performed, as far as we know, we are the first to find that JBT acts as an NF-kappa B inhibitor. However, further confirmation of this mechanism *via* more in-depth studies is still required. The application of inhibitors and positive controls, such as iguratimod, should also be considered in future research.

We here applied network pharmacology to identify signaling pathways associated with RA. The NF-kappa B pathway was shown to be activated by many stimuli, including TNF-α (Wen et al., 2018). P65 participates in the classical NF-kappa B pathway. The inhibition of p65 phosphorylation was proven to suppress the pathogenesis of RA (Roman-Blas and Jimenez, 2006; Kang et al., 2018), as JBT did in this study. In the meantime, the effect inhibiting p38-MAPK was found. As a detectable component of JBT, gentiopicroside also exhibited a potent protective effect on the IL-1β-induced inflammatory response and the release of MMPs in rat articular chondrocytes by the p38, ERK, and JNK pathways (Zhao et al., 2015). Chlorogenic acid was reported to reverse IL-1β-induced increases in IL-6, MMP-13, and COX-2/PGE2 production in human SW1353 chondrocytes, partially *via* the p65 NF-kappa B signaling pathway (Liu et al., 2017), and to induce apoptosis to inhibit the inflammatory proliferation of IL-6-induced FLSs through modulating the activation of the JAK/STAT and NF-kappa B signaling pathways (Lou et al., 2016). Moreover, isoliquiritigenin treatment inhibited IL-1β-induced MMP production and NF-kappa B activation both *in vitro* and *in vivo* (Zhang et al., 2018). Isoliquiritigenin was reported to suppress IL-1β-induced apoptosis and inflammation in chondrocyte-like ATDC5 cells by inhibiting NF-kappa B and to exert chondroprotective effects on a mouse model of anterior cruciate ligament transection (Ji et al., 2017). Xanthotoxin inhibited the expression of Runx2 and MMP-13 by reducing the activation of p38-MAPK (Cao et al., 2017). In contrast to previous studies, this study involved the first time that both HPLC–Q-TOF and network pharmacology have been applied to detect the effects of this classical herbal compound and determine the mechanisms involved. However, further studies are needed to clarify the pharmacokinetics of JBT, so as to provide references for clinical indications and the optimal dose. In the process of empirical revision, there are too many vague factors in this TCM compounds, and even the proportion of the compounds themselves needs to be optimized. Therefore, we must firstly prove the effectiveness of JBT *in vitro* and *in vivo* studies. This is the foundation for all proceeding research. Therefore, we tested the active ingredients using HPLC, with the aim of finding possible targets of JBT. In this way the follow-up work could be guaranteed. In future work, we also plan to continue to conduct experiments to explore other signal pathways by network pharmacology.

In conclusion, this study indicates that JBT could markedly ameliorate synovial inflammation and joint destruction in CIA and TNF-Tg mice. The NF-kappa B pathways were found to be closely involved in the inhibitory effects.

## Data Availability Statement

The raw data supporting the conclusions of this article will be made available by the authors, without undue reservation, to any qualified researcher.

## Ethics Statement

The animal study was reviewed and approved by Longhua Hospital - Animal Ethics Committee.

## Author Contributions

QL, HX, QS, and YW conceptualized and planned the experiments. TW and QJ performed most of the experiments and completed the original draft. TC completed the network pharmacological analysis. HY completed HPLC–Q-TOF. XL completed experiments on osteoclasts. XT and HX analyzed the data. YL and YZ raised the animals and assessed the symptoms of arthritis. CH provided guidance for target prediction. All authors were involved in the writing and critical review of the manuscript and approved its final version.

## Funding

This work was sponsored by research grants from the National Natural Science Foundation (81822050, 81920108032, and 81673990 to QL, 81873321 to HX), Leading Medical Talents in Shanghai (2019LJ02 to QL), Dawn Plan of Shanghai Municipal Education Commission (19SG39 to QL), Shanghai TCM Medical Center of Chronic Disease (2017ZZ01010 to YW), The Program for Innovative Research Team of the Ministry of Science and Technology of China (2015RA4002 to YW), Research Project of Shanghai Science and Technology Commission (17401971100 to QL), “Innovation Team” development projects (IRT1270 to YW), and Three Years Action to Accelerate the Development of Traditional Chinese Medicine Plan [ZY(2018-2020)-CCCX-3003 to YW].

## Conflict of Interest

The authors declare that the research was conducted in the absence of any commercial or financial relationships that could be construed as a potential conflict of interest.
