# Supplementary figures and images for: Alleviation of Synovial Inflammation of Juanbi-Tang on Collagen-Induced Arthritis and TNF-Tg Mice Model
Source: Front Pharmacol. 2020 Feb 14;11:45. doi: 10.3389/fphar.2020.00045 (PMC7033619; doi:10.3389/fphar.2020.00045)

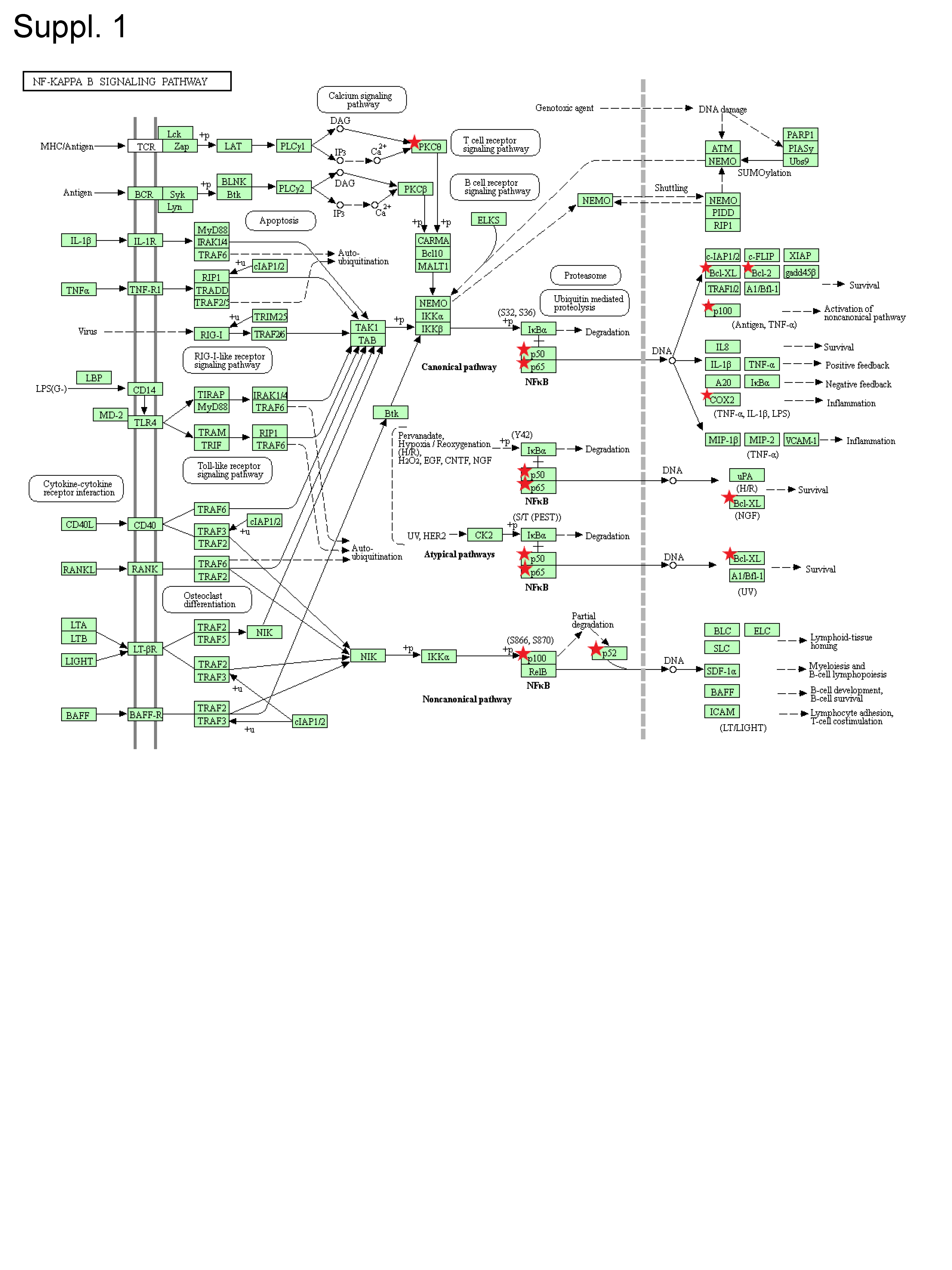

Supplement: Supplementary file 1 [file Image_1.tif]

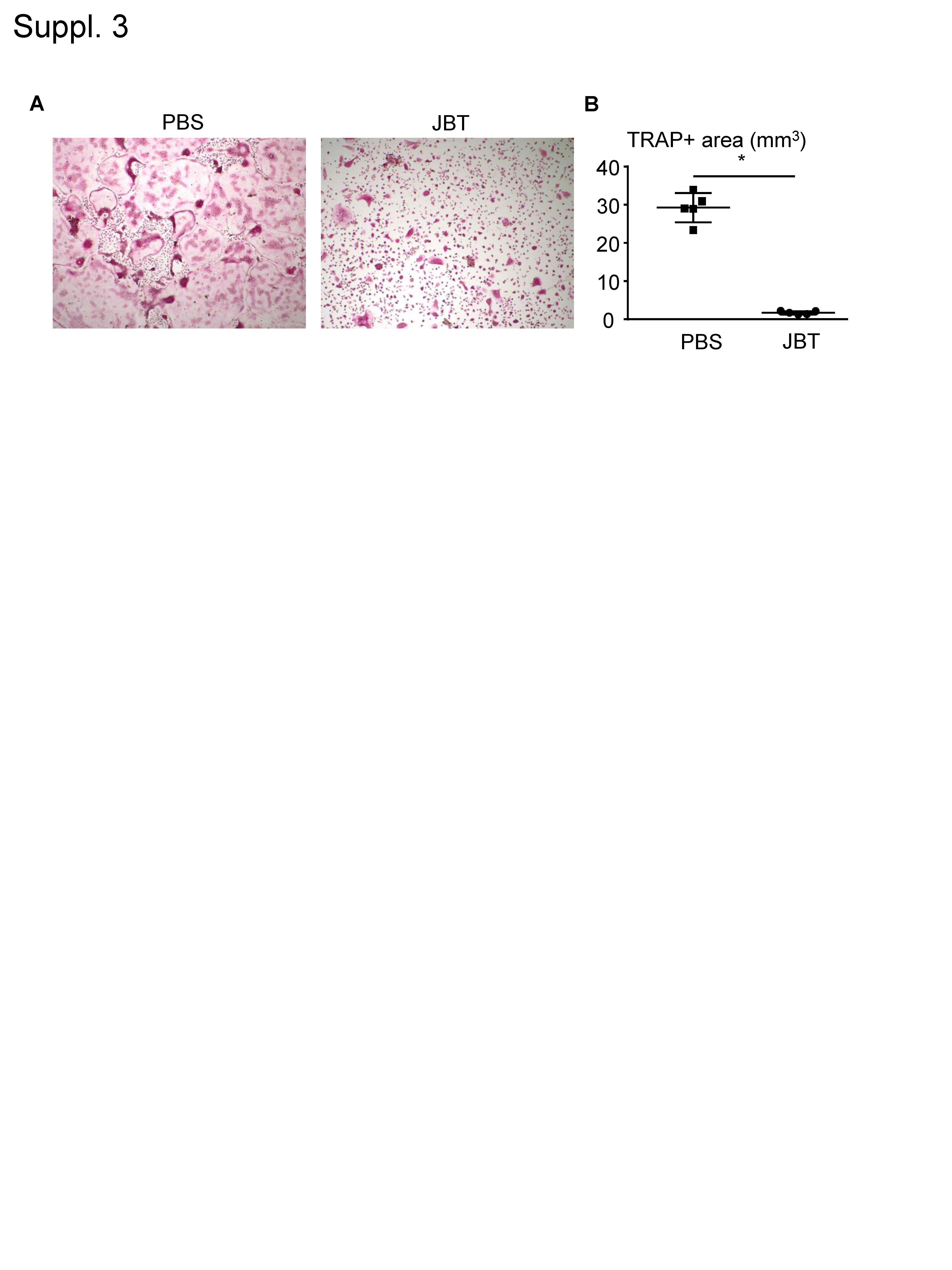

Supplement: Supplementary file 3 [file Image_3.tif]

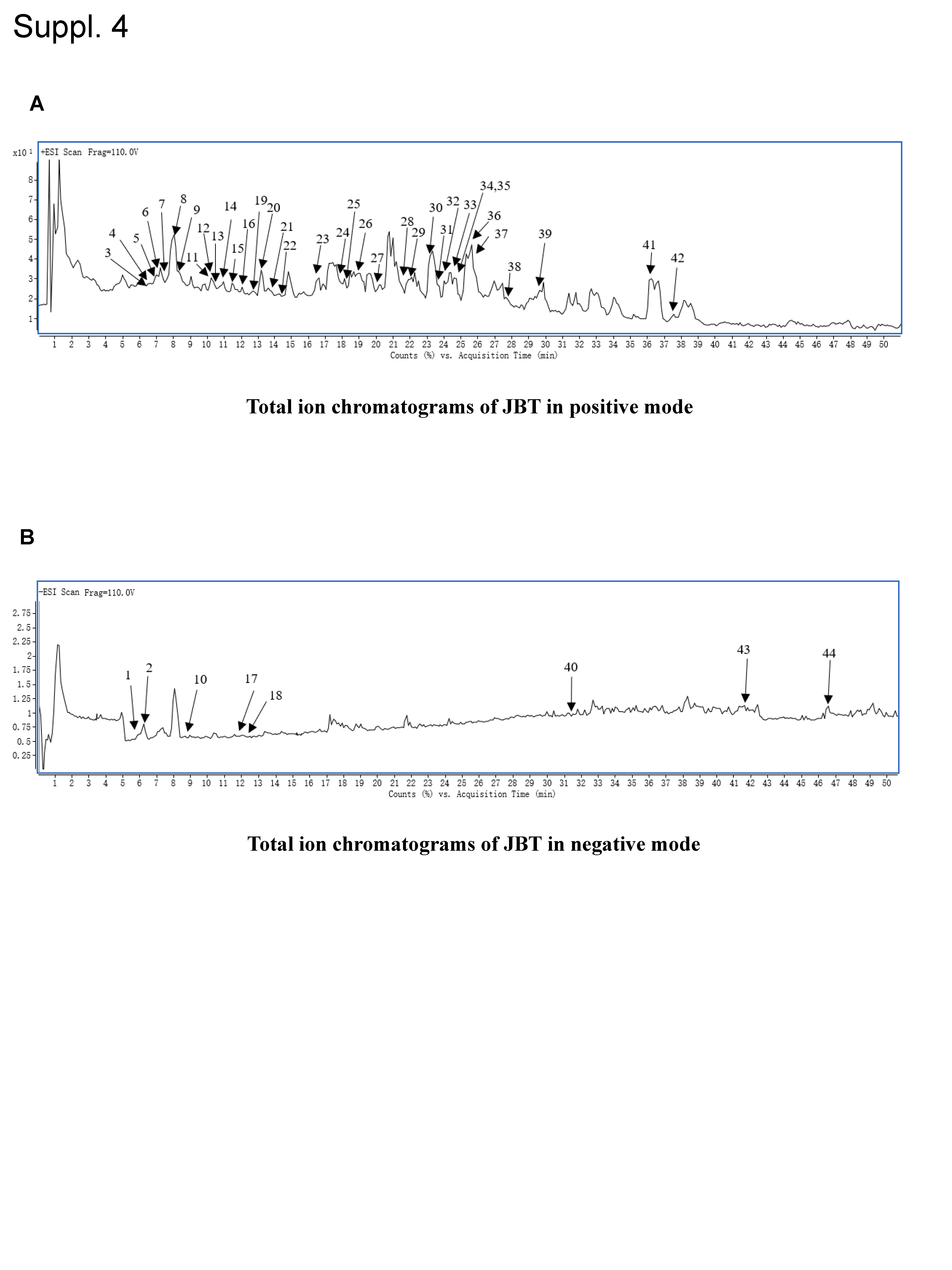

Supplement: Supplementary file 4 [file Image_4.tif]

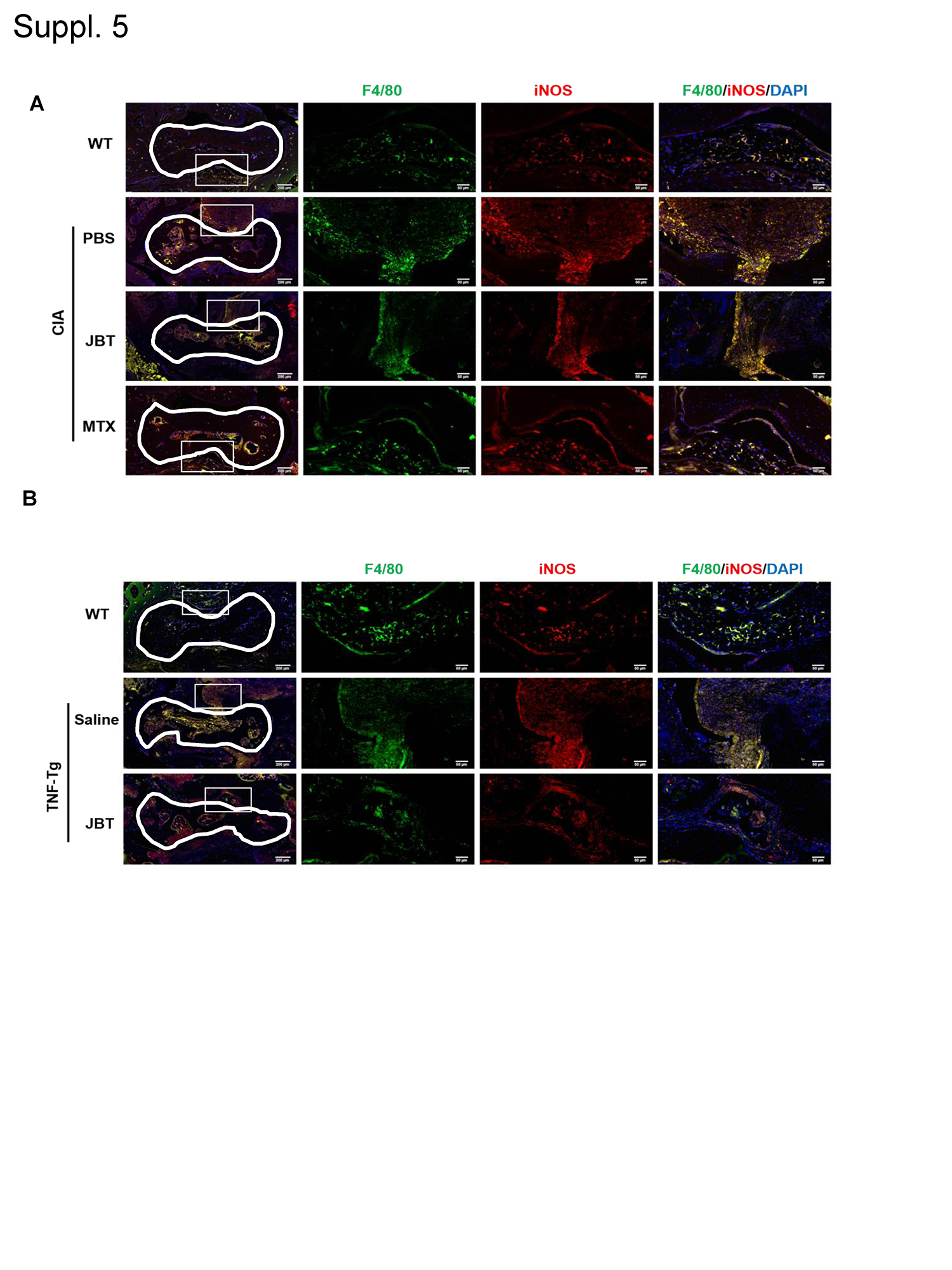

Supplement: Supplementary file 5 [file Image_5.tif]

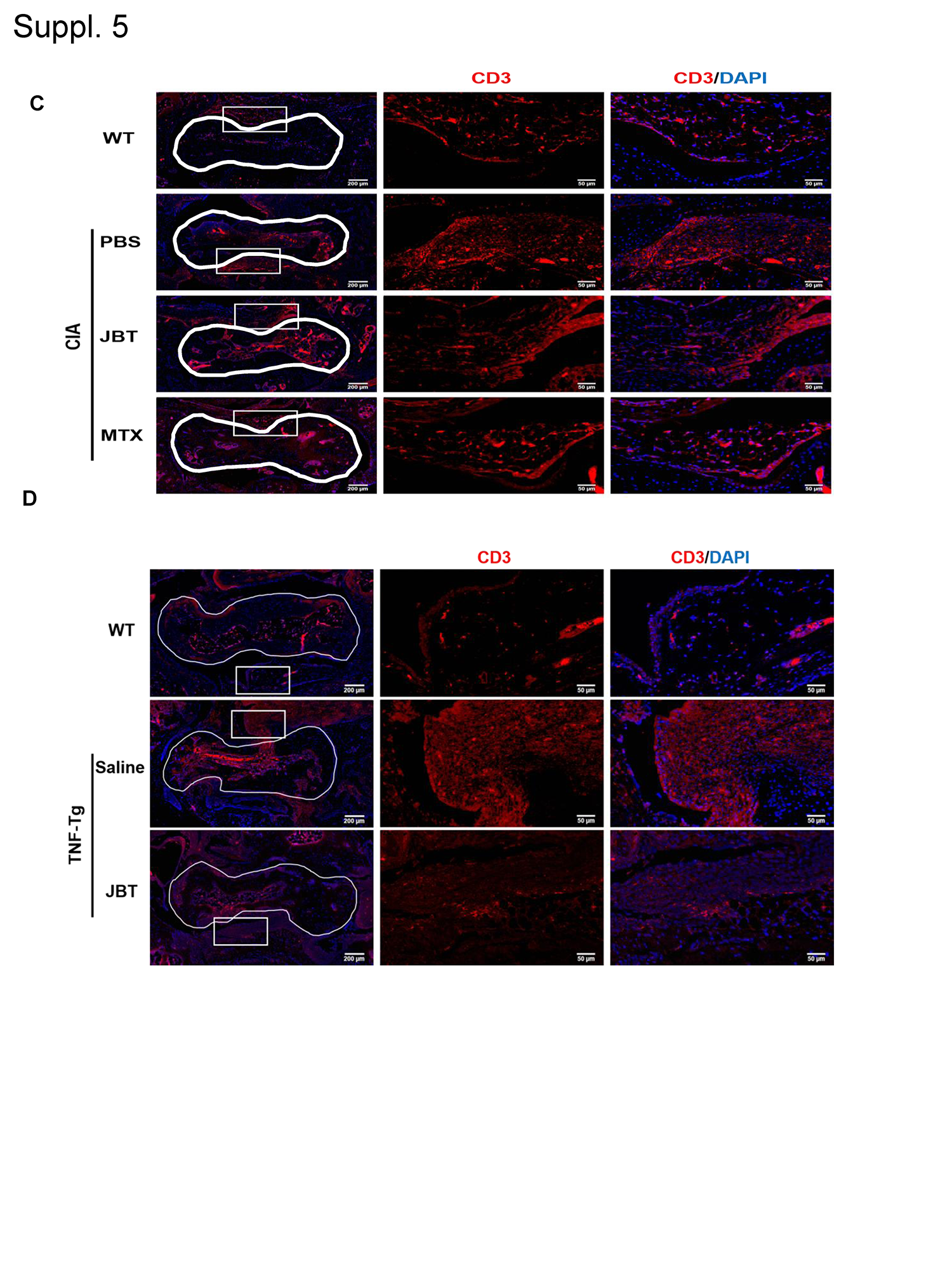

Supplement: Supplementary file 6 [file Image_6.tif]
